# Keeping Pace With 21^st^ Century Healthcare: A Framework for Telehealth Research, Practice, and Program Evaluation in Occupational Therapy

**DOI:** 10.5195/ijt.2021.6379

**Published:** 2021-06-22

**Authors:** Lauren M. Little, Kristen A Pickett, Rachel Proffitt, Jana Cason

**Affiliations:** 1 Rush University, Chicago, IL, USA; 2 University Of Wisconsin-Madison, Madison, WI, USA; 3 University Of Missouri, Columbia, MO, USA; 4 Spalding University, Louisville, KY, USA

**Keywords:** Occupational therapy, PACE Framework, Program evaluation, Research, Telehealth

## Abstract

The use of telehealth to deliver occupational therapy services rapidly expanded during the COVID-19 pandemic. There are frameworks to evaluate services delivered through telehealth; however, none are specific to occupational therapy. Therefore, occupational therapy would benefit from a framework to systematically evaluate components of telehealth service delivery and build evidence to demonstrate the distinct value of occupational therapy. The PACE Framework outlines four priority domains to address areas of need: (1) **P**opulation and Health Outcomes; (2) **A**ccess for All Clients; (3) **C**osts and Cost Effectiveness; and (4) **E**xperiences of Clients and Occupational Therapy Practitioners. This article describes the development and expert reviewer evaluation of the PACE Framework. In addition, the PACE Framework's domains, subdomains, and outcome measure examples are described along with future directions for implementation in occupational therapy research, practice, and program evaluation.

The use of telehealth to deliver occupational therapy services rapidly expanded due to the COVID-19 pandemic as practitioners in community-based settings, early intervention, hospitals, nursing homes, outpatient clinics, and schools pivoted to using telehealth to serve clients. The American Occupational Therapy Association (AOTA, 2018) defined telehealth as the “application of evaluative, consultative, preventative, and therapeutic services delivered through information and communication technology (p. 1).” Before and during the pandemic, AOTA was active in educating occupational therapy practitioners (occupational therapists and occupational therapy assistants) about telehealth through practice guidance and position papers (AOTA, 2005, 2010, 2013, 2018, 2021). Additionally, research had demonstrated the efficacy, client satisfaction, and potential cost savings of occupational therapy services delivered through telehealth (for reviews see Nissen & Serwe, 2018; Nobakht et al., 2017). However, lack of payment structures and associated decreased system and clinician utilization of telehealth limited its use in occupational therapy practice until the pandemic. The pandemic emphasized the need for quality, evidence-based approaches to telehealth delivery, and offered significant opportunities to collect and analyze data related to the implementation and outcomes of telehealth. Occupational therapy could benefit from a framework from which to draw and organize the data and guide future research and program evaluation.

There are existing frameworks for the evaluation and measurement of healthcare services delivered through telehealth. The National Quality Forum (2017) outlined four domains of measurement in telehealth: access to care, financial impact and/or cost, experience, and effectiveness. The Institute of Health (Berwick et al., 2008; Bodenheimer & Sinsky, 2014) described four pillars of telehealth, including the patient care experience, population health, affordability, and improved work-life for the practitioner. Recently, the American Academy of Pediatrics (Chuo et al., 2020) outlined four domains of measurement for pediatric focused care delivered through telehealth: health outcomes; health delivery (e.g., quality and cost); experience; and program implementation and key performance indicators. These frameworks share similarities; however, no existing framework captures the distinct measurement and evaluation needs of occupational therapy services and programs delivered through telehealth. Telehealth offers only a tool through which occupational therapy services are delivered, such a framework would encompass many concepts and ideas that are *profession specific* but not necessarily different when approached through telehealth versus in-person service delivery.

The occupational therapy profession would benefit from a framework that serves as a resource to support evaluation of evidence-based telehealth practices. The purpose of this article is to describe the PACE Framework for measurement and evaluation of occupational therapy services delivered through telehealth. We draw from existing frameworks of telehealth measurement to propose four domains that are priorities for occupational therapy: (1) **P**opulation and Health Outcomes; (2) **A**ccess for **A**ll Clients; (3) **C**osts and Cost Effectiveness; and (4) **E**xperiences of Clients and Occupational Therapy Practitioners.

## METHOD

### TELEHEALTH PLANNING GRANT COLLECTIVE

In October 2020, the American Occupational Therapy Foundation (AOTF) convened a Planning Grant Collective titled “Stimulating Research to Advance Evidence-Based Applications of Telehealth in Occupational Therapy.” This event led to the identification of several areas of telehealth research and practice. These areas were described in a publication with outcomes from that meeting (Proffitt et al., 2021). Existing telehealth frameworks were consulted in an attempt to: (a) examine the need for a new framework to collect and analyze data related to telehealth implementation, (b) capture the distinct value of occupational therapy, and (c) identify outcomes of telehealth across occupational therapy settings and populations. This process resulted in the development of the PACE Framework. Following the AOTF 2020 Planning Grant Collective, members of the AOTF Planning Grant Collective Organizing Committee continued to meet to conceptualize the PACE Framework, outline a process and agenda for expert stakeholder review, and analyze and integrate stakeholder feedback.

### OCCUPATIONAL THERAPY STAKEHOLDER EVALUATION

Prior to inviting stakeholder review of the PACE framework, the Institutional Review Board granted exempt status to the review process. We invited nine stakeholders with various backgrounds in occupational therapy practice and research and representation across practice settings (e.g., outpatient rehabilitation, early intervention), to review the PACE Framework and provide feedback. Specifically, we asked the stakeholders to use a sliding scale of 1–100 to rate the (1) clarity of the PACE Framework (0=not clear at all to 100=exceptionally clear), (2) utility of the PACE Framework (0=not useful at all to 100=exceptionally useful), (3) content of the PACE Framework (0=critical components are missing to 100=all critical components are well addressed), (4) distinctness of each construct (0=not distinct at all to 100=constructs are completely distinct), (5) alignment of outcomes within each construct (0=not aligned to 100=exceptionally aligned), and (6) possibility of implementation of the PACE Framework (0=I would not recommend PACE Framework to a colleague to 100=I would highly recommend the PACE Framework to a colleague). We also asked stakeholders four open-ended questions focused on general feedback, inclusion of additional constructs, outcomes or measures, and the ways in which the PACE Framework may be implemented in their respective practice and/or research settings.

### DATA ANALYSIS AND FRAMEWORK REFINEMENT

We used descriptive statistics to examine quantitative stakeholder responses (n=8; one stakeholder did not respond) and compiled qualitative responses for each question to refine the framework. We used content analysis (Elo & Kyngäs, 2008) to examine qualitative responses. Continuous discussion among the authors, along with integration of quantitative and qualitative stakeholder feedback, led to revisions that assured that the PACE Framework presented in this article captured domains, outcomes, and outcome measures related to occupational therapy. Quantitative ratings from the expert review panel are shown in Table 1.

**Table 1:** Quantitative Ratings from the Expert Review Panel

Item	Mean (SD)	Range
Clarity	82.88 (10.76)	71–100
Utility	81.13 (16.58)	50–100
Content	87.25 (6.43)	75–93
Distinctness	83.75 (22.51)	30–100
Alignment	84.14 (10.64)	68–95
Possibility of Implementation	83.88 (11.76)	66–100

Based on content analysis of qualitative responses, we discussed how to integrate stakeholder feedback within the PACE Framework. Qualitative comments that resulted in edits to the PACE Framework are shown in Table 2. First, in response to qualitative comments on general impressions and feedback, we added and removed numerous measurement tools to have a balance of adult and pediatric populations. Second, in response to suggestions about missing constructs, we emphasized the link between the primary domain of *Access for All Clients s* as it may be related to social determinants of health (see Results). We also emphasized that outcomes may include reported measures (e.g., occupational therapy practitioner, client, caregiver) and/or evidence from documentation or medical records. Third, we responded to reviewer suggestions about missing outcome measures by adding suggested assessments. We removed any condition specific outcome measures because researchers and/or practitioners are equipped with such knowledge of assessments specific to their contexts (e.g., stroke versus autism spectrum disorder specific measures). Fourth, with regard to envisioned use, we emphasized that the PACE Framework may be used to support evidence-based occupational therapy services, and includes both outcome and process measures.

**Table 2:** Qualitative Comments that Resulted in Edits to the PACE Framework

Survey Question	Expert Reviewer Comments	Resulting Edits
What are your overall impressions and general feedback?	“*More adult outcome tools and examples are needed*” “*Very comprehensive*” “*The PACE Framework is an excellent tool to use to guide telehealth research and program evaluation*” “*Having a reminder will foster better quality”*	More balanced outcome measures that represented pediatrics and adult populations were included.
Is the PACE Framework missing any constructs?	“*Perhaps need to look at how SDOH [social determinants of health] affect implementation*” “*Consider separating client reported outcomes and practitioner reported outcomes*”	In the Results section, we included increased supporting text to ensure that social determinants of health (SDOH) are considered within the *Access* construct. We also specified why client and practitioner reported outcomes were included, and added suggested outcome measures.
Is the PACE Framework missing specific outcome measures?	“*Days absent from school/truancy/attendance at community support activities*” “*Numerous specific assessments were recommended for +/− (i.e. “cancer or lymphedema QOL [quality of life] scales*)” “*Well-being could be expanded to include a few common caregiver burden scales*”	We included an explanation for not including diagnosis-specific assessments in the text and added suggested scales.
How do you envision implementing the PACE Framework?	“*…I think being clear about what is measuring telehealth as a delivery method (such as access and cost, satisfaction) versus outcomes of the intervention (participation, occupational performance) versus processes that are enhanced by telehealth (e.g., collaboration, coordination) would be helpful*.”	In response to the comment, we emphasized that the PACE Framework includes both process measures and outcome measures throughout the text.

### OVERVIEW OF THE PACE FRAMEWORK

The PACE Framework is comprised of four domains: (1) **P**opulation and Health Outcomes; (2) **A**ccess for All Clients; (3) **C**osts and Cost Effectiveness; and (4) **E**xperiences of Clients and Occupational Therapy Practitioners. Within each domain, we included a number of possible outcomes and associated operational definitions that aligned with AOTA's Occupational Therapy Practice Framework – 4^th^ Edition (2020d) and/or the World Health Organization (2001) when possible. The PACE Framework also includes measurable subdomains within outcomes, as many outcomes are broad in nature and may be broken into measurable components. We propose that the four domains of the PACE Framework and associated areas of specific measurement (see Table 3) can be used to capture the distinct value of occupational therapy delivered across settings and populations. The PACE Framework is unique from other telehealth measurement and evaluation guidelines, as we aligned telehealth measurement and evaluation domains with the specific needs of occupational therapy.

**Table 3:** The PACE Framework

Population and Health Outcomes		
Outcome	Operational Definition and Measurable Sub-Domains	Examples of Outcome Measures
Care coordination	Policies and practices that create coherent and timely client-centered care both within and across care settings and over time. Examples include: Communication between team members Timing and support of transition between care (e.g., acute care to in-patient rehab; early intervention to early childhood) Link to community resources	*Assessment of Interprofessional Team Collaboration Scale* (Orchard et al., 2012) *Interprofessionalism Assessment* (Frost et al., 2019) *Interdisciplinary Team Process and Performance Survey* (Temkin-Greener et al., 2004) Length of time for transition care Survey of client perceptions of quality and timeliness of care coordination
Health promotion	“Process of enabling people to increase control over, and to improve, their health. To reach a state of complete physical, mental, and social well-being, an individual or group must be able to identify and realize aspirations, to satisfy needs, and to change or cope with the environment” (World Health Organization, 1986). Examples include: Population health promotion, focused on communities and factors that influence their health Group health promotion, focused on health and engagement (e.g., engagement in leisure among older adults, reduction in bullying at schools) Individual health promotion	*Groups & Populations* Healthy People 2030 Leading Health Indicators (U.S. Department of Health and Human Services, n.d.) Population measurement: Patient-Reported Outcomes Measurement Information System PROMIS®, www.nihpromis.org (Northwestern University, 2021a) Reduction in health disparities Promotion of healthy living practices *Individuals* Healthcare utilization Measures of health status Changes in modifiable health risk factors Frequency of participation in health promotion activities
Occupational performance	“Accomplishment of the selected occupation resulting from the dynamic transaction among the client, their contexts, and the occupation” (AOTA, 2020d, p. 8). Examples include: Occupational performance Activities of daily living (ADLs) Instrumental activities of daily living (IADLs) Health management Rest and sleep Education Work Play & leisure Social participation Performance patterns Performance skills Client factors	*Canadian Occupational Performance Measure* (Law et al., 1990) *Montreal Cognitive Assessment* (Nasreddine et al., 2005) NIH Toolbox for Assessment of Neurological and Behavioral Function (Northwestern University, 2021b) *Occupational Circumstances Assessment Interview and Rating Scale* (Forsyth et al., 2005) *Occupational Performance History Interview* (Kielhofner et al., 2001) *Occupational Self-Assessment* (Baron et al., 2002) *Pediatric Evaluation of Disability Inventory – Computer Adaptive Testing* (Dumas et al., 2015) *Sensory Processing Measure* (Parham et al., 2007) *Sensory Profile-2* (SP-2; Dunn, 2014)
Participation	“Involvement in a life situation” (World Health Organization, 2001, p. 10). Examples include: Client satisfaction, enjoyment, and/or frequency of engagement in meaningful occupations and everyday activities	*Assessment of Preschool Children's Participation* (Law et al., 2012) *Canadian Occupational Performance Measure* (Law et al., 1990) *Community Participation Indicators* (Heinemann, 2010) *Goal Attainment Scaling* (Kiresuk & Sherman, 1968) *School Function Assessment* (Coster et al., 1998) *The Child and Adolescent Scale of Participation* (Bedell, 2011)
Prevention	“Education or health promotion efforts designed to identify, reduce, or prevent the onset and decrease the incidence of unhealthy conditions, risk factors, diseases, or injuries” (American Occupational Therapy Association, 2020d, p. 81). Examples include: Considerations of how occupational therapy delivered through telehealth influences health and developmental outcomes, possibly decreasing need for more intensive care later in life Prevention-focused program process measure	Safe at Home Checklist (AOTA, n.d.-c) Analysis of data related to: # of injuries, rate of absenteeism related to injury # of falls post implementation of fall prevention programming # of hospitalizations post prevention-focused occupational therapy intervention Developmental and academic outcomes among children Home safety and accessibility for fall prevention among older adults
Quality of life	“Dynamic appraisal of the client's life satisfaction (perceptions of progress toward goals), hope (real or perceived belief that one can move toward a goal through selected pathways), self-concept (composite of beliefs and feelings about oneself), health and functioning (e.g., health status, self-care capabilities), and socioeconomic factors (e.g., vocation, education, income; adapted from Radomski, 1995)” (AOTA, 2020d, p. 66). Examples include: Nutrition Stress Quality of education Economic conditions Social engagement Leisure/recreation participation	*Global Quality of Life Scale* (Hyland & Sodergren, 1996) *Health-Related Quality of Life Questionnaire* (CDC, 2000) *McGill Quality of Life Questionnaire — Expanded* (Cohen et al., 2019) *Health-related QOL PedsQL* (Varni et al., 1999) *Short Form 36 Questionnaire* (Rand, n.d.) *The Quality of Life Scale* (Flanagan, 1978) *World Health Organization Quality of Life Instrument* (WHO, 2004)
Role competence	“Ability to effectively meet the demands of the roles in which one engages” (AOTA, 2020d, p. 67). Examples include: Self-efficacy, satisfaction, prioritization, and motivation related to life roles	*Parenting Sense of Competence Scale* (Ohan et al., 2000) *Perceived Maternal Parenting Self-Efficacy Scale* (Barnes & Adamson-Macedo, 2007) *Role Checklist V3* (Scott, 2019) *Self-Management Self-Test* (Wehmeyer et al., 2019)
Self-Advocacy	“Advocacy for oneself, including making one's own decisions about life, learning how to obtain information to gain an understanding about issues of personal interest or importance, developing a network of support, knowing one's rights and responsibilities, reaching out to others when in need of assistance, and learning about self-determination” (AOTA, 2020d, p.83). Examples include: Behavioral autonomy Self-regulated behavior Psychological empowerment Self-realization	*Daily Living Self-Efficacy Scale* (Maujean et al., 2014) *General Self-Efficacy Scale* (Schwarzer & Jerusalem, 1995) *Falls Self-Efficacy Scale* (Tinetti et al., 1990) *Patient Activation Measure* (Hibbard et al., 2004) *The Arc's Self-Determination Scale* (Wehmeyer, 1999) *The Arc's Self-Determination Scale-Adolescent Version* (Wehmeyer & Kelchner, 1995)
Well-being	“Contentment with one's health, self-esteem, sense of belonging, security, and opportunities for self-determination, meaning, roles, and helping others” (AOTA, 2020d, p. 67). “A general term encompassing the total universe of human life domains, including physical, mental, and social aspects, that make up what can be called a ‘good life’” (World Health Organization, 2006, p. 211). Examples include: Sense of self-efficacy, satisfaction, stress, and burden associated with caregiving	*Caregiver* Life Balance Inventory (Matuska, 2012) WHO-Five Well-Being Index (WHO-5), WHO-Ten Well-Being Index (WHO-10) (World Health Organization, 1998) Zarit Burden Interview (Zarit et al., 1980) *Client* *Life Balance Inventory* (Matuska, 2012) OECD Guidelines on Measuring Subjective Well-being *Student Life Satisfaction Scale* (Huebner, 1991) NIH Toolbox® (Northwestern University, 2021b) Subjective well-being measures
**Access for All Clients**		
Diversity, equity and inclusion [This topic merits extensive content, which is beyond the scope of this article and must be fully addressed in future work.]	In accord with AOTA's commitment to diversity, equity, and inclusion (AOTA, 2020b) and the AOTA Vision 2025 (AOTA, n.d.-a), telehealth research, practice, and policy should reflect diversity in race, ethnicity, gender, age, socio-economic status, geography, and other demographics; promote occupational justice; and be client-centered.	Outcome measures can be extracted from the following guides: AOTA's Guide to Acknowledging the Impact of Discrimination, Stigma, and Implicit Bias on Provision of Services (AOTA, 2020a) Diversity, Equity and Inclusion in Occupational Therapy, Resources and the DEI Tool Kit (AOTA, n.d.-b) Equity & Inclusion Lens Guide (Non-Profit Association of Oregon, 2018) Ford Foundation Disability Inclusion Toolkit (Ford Foundation, n.d.)
Access to technology and internet	The extent to which technology and available internet data is sufficiently available and affordable to individuals and communities. Examples include: Broadband availability and speed in communities Individuals’ access or ownership of smartphones, tablets, laptops or desktop computers. Cost of access (i.e., laptops, smartphone, internet, data)	Amount of high-speed data available per month, per individual or family County average cellular and fixed wireless download speeds (see www.NACO.org) Number of internet subscribers in a community or neighborhood (see www.Brookings.edu) Number of devices per household Point of access for internet use (e.g., home, community, school)
Availability and usability of translators	The ways in which an organization supports the availability and quality of translation services for clients to access services. Examples include: The range and number of translation services offered at various entry points into occupational therapy treatment as well as client reported satisfaction and acceptability of translation services.	Satisfaction surveys with ways for clients to express ways to improve language services The percent of clients/patients who have been screened for their preferred spoken language The percent of clients receiving initial assessment and intervention sessions from assessed and trained interpreters or from bilingual providers assessed for language proficiency (see Regenstein, 2007). Volume of interpreter encounters within an institution, agency, or school Wait times for interpreter availability
Availability of specialists	The extent to which telehealth extends the availability of providers with specializations and/or certifications. Examples include: Number, availability, and collaboration among occupational therapy practitioners with specializations and/or certifications	Client wait times to access providers with specialty certifications Number of sessions with specialty providers Number of sessions with collaboration between specialty providers and client's original provider Percent of telehealth providers with specialty certifications within an agency, hospital, or school
Digital health literacy	The degree to which individuals have the ability to find, understand, and use information and services to inform health-related decisions and actions for themselves and others. Examples include: Finding and consuming digital content Creating digital content Communicating and/or sharing digital content Evaluating quality and relevance of digital content	*Digital Health Literacy Instrument* (van der Vaart & Drossaert, 2017) *eHealth Literacy Assessment Toolkit* (Karnoe et al., 2018) *eHealth Literacy Questionnaire* (eHLQ; Kayser et al., 2018) *eHealth Literacy Scale* (eHEALS; Norman & Skinner, 2006) Tracking the level of support that individuals, including children, require to log on and navigate telehealth sessions
Integration and use of clients’ everyday materials	The ways in which assessment and intervention sessions use clients’ readily available materials in their natural contexts. Examples include: Using clients’ and families’ materials for assessment and intervention Any specialized materials and/or equipment that clients/families are asked to purchase to engage in the occupational therapy evaluation and/or intervention	Any documentation to prepare clients and/or families about expectations regarding upcoming sessions Documentation about what materials/intervention activities in which clients and families engaged Documentation that would reflect any “specialized” materials and/or materials that clients/families would have to purchase to complete the intervention session
Organizational digital health literacy	The degree to which organizations equitably enable individuals to find, understand, and use information and services to inform health-related decisions and actions for themselves and others (CDC, 2021). Examples include: Organizational structure, policy, and leadership supports for telehealth software that supports clear client-occupational therapy practitioner communication, and is easily navigated by occupational therapy practitioners and clients	Availability of validated assessment measures that are compatible with numerous telehealth platforms Ease of integration of assessment measures, documentation, and client communication within telehealth software Leadership support for practitioner and client training to access telehealth
Technology usability	The extent to which available technology is appropriate for telehealth access, including evaluation and intervention sessions. Examples include: Effectiveness, efficacy, and satisfaction with the device and internet quality of accessing telehealth sessions	Amount of time to log on to telehealth sessions *Computer Proficiency Questionnaire* (Boot et al., 2015) *Mobile Device Proficiency Questionnaire* (Roque & Boot, 2018) Number of internet disruptions/slow internet miscommunications during a session The amount of client assistance needed to schedule and log on to a session The extent to which the device/internet speed allows for effective communication between the client and practitioner
Scheduling ease and convenience	Client reports of scheduling ease as convenient and fitting into daily life. Examples include: Client reported ease and satisfaction with setting up and attending telehealth sessions	Availability of occupational therapy practitioners on evenings and weekends to match clients’ schedules Client satisfaction survey with questions about scheduling convenience and availability of appointments
**Cost and Cost Effectiveness**		
Client costs and cost savings	The costs and cost savings associated with accessing and attending telehealth sessions; clients may save expenses due to convenience of telehealth and/or incur costs if any additional technology or data is necessary to access telehealth sessions. Examples include: Travel considerations related to time and distance may be dependent on community setting (e.g., rural vs. urban) and client reported method of transportation	*Costs* Costs incurred by clients, including sufficient internet connectivity and technology devices to access appointments *Savings* Cost savings related to client burden reduction including: travel expenses (gas, food) time off work for travel to appointments missed work or school days childcare expenses associated with appointment public transportation costs fuel costs and costs associated with parking personal vehicle, if applicable attendance at community support activities Calculated mileage/travel distance (Note: Distance may be appropriate to measure for suburban and/or rural samples, while for urban samples, measurement strategies may be based in time, where public transportation or traffic are considered.) Clients’ report of travel distance and time with their specific method of transportation (e.g., car travel may be faster than public transit travel)
Practitioner costs and cost savings	The costs and cost savings among practitioners that result from telehealth. Examples include: Saved expenses due travel time and costs, and/or incurred costs if software, technology, or additional data is necessary to conduct telehealth sessions	Costs associated with telehealth software, multiple state licenses, internet and technology (e.g., hardware, software, peripherals) Miles from home to clinic or hospital setting Travel distance/time for therapy practitioner(s) to travel (between home, hospital, clinic(s), school(s), clients’ homes)
Relation of service utilization to long term outcomes	The degree to which costs of occupational therapy delivered through telehealth are associated with long term health and/or developmental outcomes across clients and settings. Examples include: Expenses that would likely have occurred if service was not provided (e.g., re-hospitalization, development of pressure ulcer)	Analyses using an incremental cost-effectiveness ratio (ICER) to determine if clients’ functional gains over time differ by service delivery model (e.g., in-person, hybrid, telehealth) Analyses that compare groups’ outcomes among those that receive occupational therapy by different service delivery models (e.g., in-person, hybrid, telehealth) Emergency department (ED) visit avoidance in real time and/or future Healthcare utilization, compare to a normative database Comparison of adopters to non-adopters to long term health outcomes (e.g., cohort design)
Service provision and utilization	The extent to which occupational therapy services are offered, available, and attended by clients across settings and communities.	Rate of attendance, which includes number of cancelled appointments and/or no shows The number, frequency, and length of sessions that were used to achieve a specific goal or gain in function The ratio of number, frequency, and length of sessions that are attended by clients Total number, frequency, and length of time of recommended services
**Experiences of Clients and Occupational Therapy Practitioners**		
Authentic contexts	The extent to which telehealth sessions occur within clients’ authentic contexts and address clients’ everyday activities.	Assessment results that reflect clients’ performance in their everyday environments Documentation about how everyday routines look for clients in their natural context Documentation of locations in which sessions occur Documentation of locations of both clients and occupational therapy practitioner Evidence of ecological validity of assessment approaches Potential measures of generalization of how clients/caregivers can use intervention strategies used in everyday environments
Caregiver/care partner acceptability and satisfaction	The acceptability and perceived quality of the service delivery mechanism from the perspective of the caregiver for younger clients and/or trusted supporter for older clients.	*Canadian Occupational Performance Measure* (Law et al., 1990) *Telehealth Acceptability and Satisfaction Questionnaire* (e.g., Little et al., 2018; Vismara et al., 2012) *Washing Co. Family Caregiver Satisfaction Survey* (Washington Co. Family Caregiver Support Program, n.d.)
Client acceptability and satisfaction	The perceived acceptability, value, and client attributed outcomes of telehealth delivered occupational therapy services.	*Canadian Occupational Performance Measure* (COPM; Law et al., 1990) *Telehealth Acceptability and Satisfaction Questionnaire* (adapted for clients’ self-report) (e.g., Little et al., 2018; Vismara et al., 2012) Client satisfaction influenced by perceived benefits of telehealth (e.g., saved workdays or school days, reduced travel, time, and costs associated with receiving care through telehealth) Surveys that incorporate clients’ reports of functional gain as a result of telehealth
Inclusion of care partners (caregiver/family/other)	The extent to which clients’ care supporters actively participate in and are included in the occupational therapy process (i.e., assessment, intervention, re-evaluation).	Documentation of care supporter's engagement in the session The % of time the care supporter participated in the session The % of time the practitioner engaged with the care supporter
Practitioner acceptability and satisfaction	The extent to which occupational therapy practitioners perceive that telehealth promotes wellness, reduces burnout, and is an effective mechanism to deliver assessments and interventions that meet clients’ needs and achieve evidence-based practice standards.	*Maslach Burnout Inventory* (Maslach & Jackson, 1981) *Oldenburg Burnout Inventory* (Demerouti et al., 2001) *Professional Quality of Life Measure* (Stamm, 2009) *Stanford Professional Fulfillment Index* (Trockel et al., 2018) *Telehealth Acceptability and Satisfaction Questionnaire* (adapted for practitioners’ responses) (e.g., Little et al., 2018; Vismara et al., 2012) *WHO-5 Well-Being Index* (WHO, 1998)

The PACE Framework was designed for outcome and process evaluation of evidence-based interventions delivered through telehealth. Although specific elements of the PACE Framework are applicable to in-person occupational therapy services, the entirety of the Framework was created with the intention of informing telehealth and hybrid service delivery. Therefore, the example outcome measures provided are easily administered through telehealth. Example outcome measures reflect observation, process evaluation, and self-, caregiver-, and practitioner-report, as well as evidence that may be gathered through chart and/or documentation review. There is a lack of evidence supporting administration of behavioral/performance-based assessments, and we chose not to include many of these in the PACE Framework (AOTA, 2018). The PACE Framework is shown in Table 3.

In accord with AOTA's commitment to diversity, equity, and inclusion (AOTA, 2020b) and the AOTA Vision 2025 (AOTA, n.d.-a), telehealth research, practice, and policy should reflect diversity in race, ethnicity, gender, age, socio-economic status, geography, and other demographics; promote occupational justice; and be client-centered. All programs and research projects that use telehealth must consider how to integrate measures that capture the complexities associated with uncovering inequities that may be associated with the use of telehealth and/or how telehealth may be used to overcome inequities in access. Occupational therapy researchers, practitioners, and policy-makers must consider how measurement across all domains within the PACE Framework will contribute to ongoing discourse and subsequent actions to address inequity. While a full discussion of how telehealth intersects with diversity, equity, and inclusion is beyond the scope of this article, we do suggest the following resources as examples that may guide telehealth programming and measurement across domains: (1) Diversity, Equity and Inclusion in Occupational Therapy, Resources and the DEI Tool Kit (AOTA's, n.d.-b); (2) Equity & Inclusion Lens Guide (Non-Profit Association of Oregon, 2018); (3) Disability Inclusion Toolkit (Ford Foundation, n.d.) and (4) Guide to Acknowledging the Impact of Discrimination, Stigma, and Implicit Bias on Provision of Services (AOTA, 2020a). While diversity, equity, and inclusion are relevant to all domains of the PACE Framework, potential measures are represented in the ***A**ccess for All Clients* domain. To underscore the importance of measurement and consideration of diversity, equity, and inclusion as well as to avoid a circumstance wherein consumers of the PACE Framework might overlook this critical area, it is visually represented in the ***A**ccess for All Clients* domain.

### POPULATION AND HEALTH OUTCOMES

Occupational therapy delivered through telehealth is ultimately aimed at increasing individuals’ participation in everyday activities within authentic contexts to improve health. The PACE Framework captures approaches to telehealth intervention that occur at the person, group, and population levels. Given occupational therapy's focus on participation, our measurement approaches should encompass methods of understanding the influence of telehealth on population health as well as individual client outcomes. Occupational therapy outcomes within the *Population and Health Outcomes* domain include care coordination, occupational performance, participation, prevention, promotion of health and wellness, quality of life, role competence, self-determination/self-management, and caregiver and client well-being (AOTA, 2020d). The outcomes of occupational therapy services are numerous; the purpose of the PACE Framework is to outline outcomes appropriate for telehealth service delivery. Therefore, the outcomes and associated measures in this section are limited to ones that lend themselves to telehealth (e.g., objective measures from documentation or medical records; observation; self-, practitioner-, and caregiver-report) and span age ranges, practice settings, and populations.

### ACCESS FOR ALL CLIENTS

We conceptualize the measurement and evaluation of access broadly. Access to telehealth is not limited to technology and internet, although both are vital to telehealth use. We structured the PACE Framework domain of ***A**ccess for All Clients* to align with occupational therapy's commitment to diversity, equity, and inclusion (AOTA, 2020b). Telehealth research, practice, and policy should reflect diversity across person and environmental factors, including race, ethnicity, gender, age, socio-economic status, geography, and other demographics. Programs and research projects must collect and report such data to inform approaches that promote occupational justice. Factors that directly influence access include technology usability, digital literacy, health literacy, availability and usability of translators, scheduling when convenient for clients and caregivers, availability of specialists, and intervention approaches that use everyday materials. Occupational therapy practitioners have expertise in evaluating and resolving access barriers to support clients’ participation in services delivered through telehealth.

### COSTS AND COST EFFECTIVENESS

A changing policy and payment landscape necessitate the demonstration of efficacy and value (both financial and non-financial) of occupational therapy services provided through telehealth. Components of cost and cost effectiveness associated with telehealth may include a decrease in cancelled appointments and “no shows,” and indirect cost savings for clients such as less time off work and elimination of travel to therapy appointments. In addition, occupational therapy practitioners working in settings that require travel to clients’ homes or between facilities (e.g., schools, nursing homes) also experience cost avoidance associated with decreased travel. Prevention of hospitalization/re-hospitalization, pressure ulcers, and other health-related complications are examples of cost-avoidance that may occur and factor into the value of occupational services provided through telehealth.

### EXPERIENCES OF CLIENTS AND OCCUPATIONAL THERAPY PRACTITIONERS

Telehealth is a natural fit for occupational therapy interventions aimed at increasing participation in authentic contexts through the use of occupation. Telehealth creates opportunities for providing and measuring occupational therapy services in authentic contexts, and the inclusion of caregivers during telehealth sessions changes the overall experience for the client and the occupational therapy practitioner. Measuring client, caregiver/care partner, and provider satisfaction are all essential to understanding the overall experience of telehealth. Preferences for and acceptability of all components of telehealth, such as the videoconference platform and session frequency or intensity, should be considered. Assessing the client, caregiver, and practitioner experience early and at frequent intervals will facilitate adoption and long-term use.

## DISCUSSION

The goal of occupational therapy is to facilitate participation in authentic contexts through the use of occupation. With a focus on participation and performance in everyday life, the profession of occupational therapy is well-positioned to serve as a leader in demonstrating the benefits of services delivered through telehealth. The occupational therapy profession must leverage expertise in participation, occupational performance, and authentic contexts to show the value of services delivered through telehealth regardless of population or setting.

**Figure 1:**
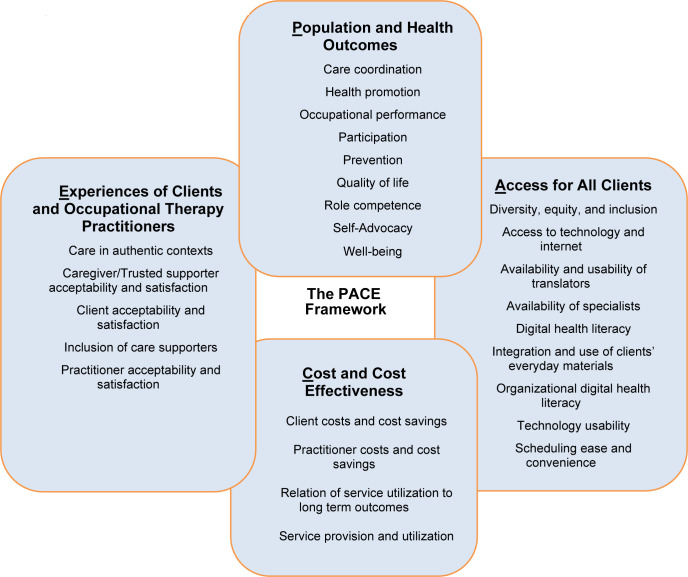
Conceptual Model of the PACE Framework

The intention of the PACE Framework is to provide a *menu* of domains, outcomes, and associated outcome measures that are possible within the context of telehealth. The PACE Framework is not meant to serve as a checklist of requirements for telehealth-based interventions; instead, we offer areas for outcome measurement, subdomains, and measurement tools to consider during the design, implementation, adoption, and dissemination of occupational therapy services delivered through telehealth. The four domains of the framework function as a Venn or logic diagram in that each domain is definable, independent from the whole, but all domains are also related to the whole and overlap in numerous areas (Figure 1).

The PACE Framework can be used to support researchers, practitioners, and policy-makers who are determining how to best capture the value of occupational therapy services delivered through telehealth. The PACE framework provides a set of telehealth measurement and evaluation domains that are priorities in occupational therapy research and practice. Researchers and occupational therapy practitioners can begin to collaborate across settings to collect data specific to outcomes and/or sub-domains that demonstrate the distinct value of services delivered through telehealth. To this end, how the framework is utilized by an end-user(s) will likely vary widely. Some may choose to focus their work on a single aspect of one component of one domain, while others may address all four domains in their design. Similarly, the PACE Framework is meant to be useful in both a prospective and retrospective manner. Prospectively, the PACE Framework is designed to help guide study or intervention design by facilitating discussion of all aspects of the occupational therapy telehealth implementation process. Retrospectively, the PACE Framework can be used to organize and analyze data from research and program evaluation. Using the PACE Framework, occupational therapy can continue to build evidence that can be shared with various stakeholders (e.g., clients, policy makers, administrators, payors, and other healthcare practitioners).

The PACE Framework shares similarities with other professions’ and organizations’ telehealth measurement frameworks (Bodenheimer & Sinsky, 2014; Chuo et al., 2020). Across practice settings and professions, frameworks outline the importance of client and provider experiences, and measure effectiveness, cost, and access. The PACE Framework, however, has three important differences that are relevant to occupational therapy delivered through telehealth. First, population health was included in the PACE Framework due to occupational therapy practitioners’ emergent role in health promotion and prevention (AOTA, 2020c). Systematic approaches to document process and outcome measures are needed for evidence-based occupational therapy programs delivered through telehealth to promote prevention, self-advocacy, and well-being for individuals and groups. Second, the PACE Framework explicitly included outcomes and example measures related to caregivers, or care partners. For many settings and populations, occupational therapy practitioners and researchers must establish partnerships with clients’ caregivers to facilitate successful access and utilization of telehealth. Instead of considering caregivers as secondary to the intervention process, the PACE Framework offers specific ways that caregivers may be recognized as essential participants in many occupational therapy interventions and measured as such.

Finally, ***A**ccess for All Clients* is considered a construct with multiple sub-domains; services must be purposefully designed and implemented to ensure access for all clients. The PACE Framework outlines sub-domains of access that must be considered, which include but are not limited to the consideration of authentic contexts, use of clients’ and families’ everyday items during telehealth sessions, and scheduling issues that have been shown to disproportionately impact clients from lower socio-economic status and minority backgrounds. Aligned with diversity, inclusion, and equity initiatives, the PACE Framework's *Access for All Clients domain* and associated sub-domains are meant to call attention to the explicit and implicit biases that create barriers to telehealth across individuals and groups. We invite researchers, practitioners, and policy makers to think broadly about access and ways to create opportunities for clients across settings and populations to use telehealth.

## CONCLUSION

The COVID-19 pandemic necessitated a shift in service delivery to telehealth across practice settings and occupational therapy practitioners adapted in response. The PACE Framework provides a means for occupational therapy practitioners and researchers to continue to build the telehealth evidence base in occupational therapy. The changing practice, policy, and payment landscape necessitates researchers and occupational therapy practitioners support best practices and demonstrate the efficacy of occupational therapy services. The PACE Framework facilitates a systematic approach to evaluating components of occupational therapy service delivery through telehealth. It is vital that occupational therapy demonstrates a concerted effort in the measurement and evaluation of services delivered though telehealth to keep PACE with 21^st^ century healthcare, meet the demands of payors moving towards value-based payments for services, and demonstrate the distinct value of occupational therapy services delivered through telehealth.
